# Observations on the Necessity of Parochial Fever Wards; with Remarks on the Extensive Spread of the Present Fever

**Published:** 1819-07-01

**Authors:** 


*Observations on the Necessity of Parochial Fever Wards; with Remarks on the extensive Spread of the present Fever.* By James Parkinson, Member of the Royal College of Surgeons. Octavo, sewed, pp. 20. London, 1819.

The policy of the measure here recommended can scarcely be disputed; but on the mode and the means of effecting it, many discrepancies of opinion will doubtless prevail.

Mr. Parkinson has discussed this important topic with much discretion and good sense, and we hope his arguments will have weight with the parochial and constituted authorities. To those who cavil at the expence of these fever wards, Mr. Parkinson replies that—"An Establishment, with eighteen beds, six for males, and twelve for females, would, it is expected, be sufficient for the paupers belonging to a parish whose population is from forty to fifty thousand persons; and the beds and the building might always be usefully employed as an infirmary, when the fever was subdued. The bedsteads should be of iron; the beds should be either of straw, or, perhaps better, of the willow shavings, the refuse of the hat-makers; and the building might be of the cheapest construction, so that perhaps the whole expence of its erection might not exceed four or five hundred pounds." P. 11. Mr. P. has drawn many affecting pictures of the wretched scenes which present themselves to the medical practitioner, in his professional walks through the habitations of sickness and poverty in this vast metropolis—the emporium of wealth and want, of wisdom and folly, of virtue and vice, and of every contrast and contrariety, frightful, flattering, and disgusting!

